# A cross-case analyses of laboratory professionals-patients interaction for patients accessing laboratory services at University of Cape Coast hospital and Ewim Polyclinic in the Cape Coast Metropolis, Ghana

**DOI:** 10.1186/s12913-021-06560-8

**Published:** 2021-05-28

**Authors:** Patrick Adu

**Affiliations:** 1 Department of Medical Laboratory Science, School of Allied Health Science, University of Cape Coast, Cape Coast, Ghana; 2 Lancaster University Management School, Lancaster, UK

**Keywords:** Laboratory testing turnaround time, Laboratory professionals-patient interactions, Laboratory test requisition, Standard operating protocols

## Abstract

**Background:**

There is scarcity of data on experiences of patients who access laboratory services during hospital visits in sub-Saharan Africa. This study sought to evaluate the depth of laboratory professionals-patient interactions during pre- and post-sampling period at two hospitals in Ghana.

**Methods:**

This study used real time observations of patient-laboratory staff interactions to collect first-hand data. Additionally, two separate sets of semi-structured questionnaires were used to collect data on the experiences of patients and laboratory professionals. Data were entered into Microsoft Excel and analysed using SPSS version 25.

**Results:**

Inadequate laboratory space is a major factor limiting adequacy of patients-laboratory professionals’ interactions. Overall, even though the laboratory professionals (93.3%) overwhelmingly agreed to the need to inform patients about the turnaround time of the respective laboratory testing, this was not routinely done. Irrespective of patients’ educational attainment, patients were poorly informed about their respective laboratory tests. Although both patients and laboratory professionals (60.0% vs 63.6% respectively) indicated that the test requester has responsibility to inform patients about their laboratory testing, only 29.1% of patients indicated having received such explanations. Furthermore, although 28.1% of patients indicated knowing the specifics of their respective test requisition, only 15% could correctly identify their requested laboratory testing.

**Conclusion:**

There is the need for standard operating protocols to standardize practitioner-patient interaction at the two facilities. Moreover, there is the need for laboratory staff-test requester engagement to clearly delineate who has what responsibilities regarding informing patients about laboratory testing.

**Supplementary Information:**

The online version contains supplementary material available at 10.1186/s12913-021-06560-8.

## Background

With evidence suggesting that many patients acquire additional infections during their time at the hospital [1, 2], patients should ideally spend the least possible time during in- and out-patient hospital visits. Though laboratorians traditionally focus on precision and accuracy of laboratory results [3, 4], clinicians additionally focus on timeliness of laboratory results [5, 6] for prompt medical decision making. Although research regarding patient turnaround time from various departments in the hospital setting abounds [7, 8], research that interrogates actual patient experiences at the medical laboratory units of hospitals is scarce. Moreover, in Ghana, the medical laboratory departments of most hospitals are usually overcrowded with many patients as they await sample taking and/or issuance of laboratory results. As these patients have differing diseases conditions, such congestion provides opportunities for cross-infections (as highlighted by the COVID-19 pandemic). These could be averted if the laboratorians adequately communicate the estimated turnaround time per a patient testing to inform patient decisions during waiting times. Additionally, as different laboratory tests require different times to complete, failure to communicate these turnaround times creates opportunities for suspicion and quarrel between the laboratorians and patients especially when some patients appear to get their results quickly while others wait for hours.

In Ghana, overwhelming majority of health system uses manual documentation systems. Thus, typically, patients are assessed by their clinicians and when necessary, are issued with laboratory test requisition form. Such patients visit the laboratory unit where laboratory professionals interact with these patients, give the necessary pre-sampling patient preparation information, before taking specimen necessary for the requested laboratory testing. When the sampling is done, the laboratory staff gives the needed post-sampling information to clients which include the estimated time when test results will be ready for pick up. Patients take the laboratory report back to the test requester to assist in clinical decision making.

This study sought to interrogate the laboratorian-patient interactions to identify any gaps in communication with potential to negatively affect the experiences of patients accessing laboratory services at the University of Cape Coast (UCC) hospital and Ewim polyclinic. This study focused on the depth of laboratory professional (LP)-patient communication to try to understand what exactly constitutes the existing pre-sampling and post-sampling communication standards. The overarching aim was to generate empirical data that could shed light on what actually prevails during LP-patient interaction, particularly, the adequacy of pre- and post-sampling information given to patients. Additionally, this was an opportunity to appraise the professional practice of LPs to unearth potential avenues for capacity building. Inadequate pre-sampling patient preparation has the potential to introduce irreversible errors into patient laboratory results as improperly acquired patient sample will lead to artefactual results that may either lead to wrong medical diagnoses or delay in diagnoses. This is a critical aspect of patient care considering that about 70% of all medical decisions are based on laboratory results [9]. Therefore, failure to assure quality of this aspect of patient care will inadvertently lead to incorrect medical decisions.

## Methods

### Study period

This study was undertaken from June, 2020 to September, 2020 (a total of 16 weeks). The first 6 weeks were used for the observation of LP-patient interactions; 3 weeks each at UCC and Ewim Polyclinic laboratories respectively. The next 8 weeks were used for questionnaire administration; 4 weeks each at UCC and Ewim Polyclinic laboratories respectively. The remaining 2 weeks were used for questionnaire administration to the laboratory professionals. Although the study was originally scheduled to be undertaken from March, 2020 to September, 2020, the COVID-19 pandemic and the subsequent lockdown delayed and limited the sampling period as well as sample size.

### Study setting

The University of Cape Coast (UCC) hospital was initially established by UCC to attend to health conditions of its students and staff. However, individuals in the surrounding communities also access the hospital. Majority of the hospital attendants are however, tertiary students. All units of the hospital run a 24-h OPD and in-patient services. On average, the laboratory unit attends to 94 patients (both OPD and in-patients) per day, with peak patient turn up between 7:30 am to 12:00 noon. The typical patient numbers in one of the weeks of sampling in the course of this study was 93, 86, 109, 81, 100 for Monday, Tuesday, Wednesday, Thursday, and Friday respectively.

Ewim Polyclinic however, is a community hospital established by the Cape Coast Metropolitan assembly to serve the Ewim, Brafoyaw, Moree, Fourth-ridge, and the surrounding towns; this hospital thus serve a wide range of patients with varying educational background. Although the hospital runs a 24-h services, the laboratory unit is on call after 8:00 pm each day. The laboratory unit averagely attends to 62 patients per day. The typical patient numbers in one of the weeks of sampling in the course of this study was 60, 67, 56, 52, and 74 for Monday, Tuesday, Wednesday, Thursday, and Friday respectively.

Records from the UCC laboratory registry indicated that the number of attendees over the period of patient questionnaire administration was 2345 (both out- and in-patients) compared to 1545 (both out- and in-patients) at Ewim Polyclinic laboratory. Even though the two hospitals run a 24-h services, the data collection was spread between the morning and afternoon shifts only as well as for only out-patients; there were no data collection during the night shifts. Also, there were no data collection during weekends. Overall, the patients were not eager to answer questionnaire intimating that the journey through the hospital systems were too stressful in itself. Additionally, others were hesitant to handle paper in a COVID-19 pandemic era. Further attempts to get either email address or phone contact to allow questionnaire administration online were not entertained by patients. This low patronage is an important limitation to the study.

### Study design

This study sequentially collected data from three different perspectives; researcher observations of actual LP–patient interactions, patient-administered questionnaires responses and LP-administered questionnaires. Patient questionnaires were administered only after the observational data collection were finished at the two hospitals. Laboratory staff questionnaires were also administered only after the all the patient-perspective data had been collected. This thus provided a methodological triangulation to assure the validity of the findings. By complementing questionnaire data with observational data, the researcher was able to reconcile what appeared to be apparent discrepancies between patients and laboratory practitioners’ questionnaire responses. Additionally, the findings of the observational data collection informed the specific items on the patient and laboratory scientist questionnaires.

Moreover, the data collection was purposely sequential because the observations were firstly undertaken at both hospitals. This was then followed by the administration of patients’ questionnaires as the second step. It was only after collecting patient data from both hospitals that questionnaires were administered to laboratory practitioners to collect practitioner-perspective data. The rational for this intentional sequential data collection was to prevent systematic error due to bias. For example, if laboratory professional knew that the exact data being collected involved how much information was provided the patients at the pre-sampling phase as well as the information relayed to the patients during post-sampling period, there is every likelihood that the laboratory personnel would have modified their practices during the duration of the study to the extent that the results would have been artefactual. Moreover, by ensuring that the data collection at the two hospitals were undertaken by the researcher who repeated each stage in the two hospitals before proceeding to roll out the next phase, not only was the researcher able to prevent information bias but also prevented the possibility of information filtering through from one hospital to the other to influence responses by the laboratory professionals.

### Observational data collection

**Fig. 1:**
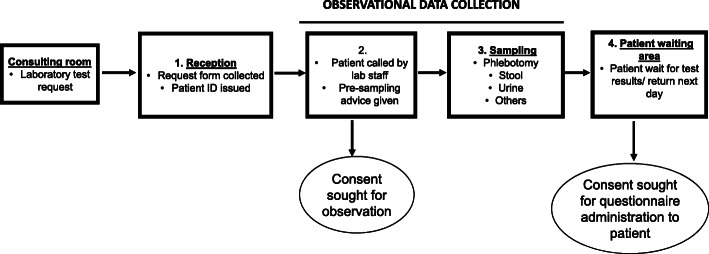
A schematic of patient journey through the laboratory system in the two facilities. The numbered blocks (1–4) represent the main points of laboratory professional-patient interactions within the laboratory department. The observational data collection covered steps 2 and 3

In this arm of the study, the researcher recorded actual patient-laboratory professional interaction verbatim. The observations were undertaken from June 1 – July 10, 2020. As the hospitals where the study was undertaken use manual documentation systems, patients take their laboratory requisition form (from their clinicians) to the reception of the laboratory; these are registered at the reception and issued with unique identification number. Patients are subsequently called to the sampling area in the laboratory (*see* Fig. 1*for patient journey through the laboratory*). For patient tests requiring blood samples, patients are referred to the phlebotomy area; otherwise, patients are issued with relevant containers and advised on how to collect urine and/or stool samples as may be required. After sampling, patients are given post-sampling information. The observations covered reception of the laboratory requisition form, pre-sampling information provided to patients, actual interactions that took place during sample reception and/or phlebotomy sessions as well as post-sampling information provided to the patients. The researcher interacted with and sought the consent from patients when they entered the sampling area; only those who gave verbal consent were included in this arm of the study. Convenience sampling technique was employed to recruit consecutively consenting individuals (18 years and above). Additionally, the specific laboratory tests requested for each patient was recorded by the researcher to allow comparison of the tests with the post-sampling information provided to the patients. The duration of a particular LP-patient interaction session depended on the nature of sample required per patient. All observational data collection was undertaken by the researcher.

### Semi-structured questionnaires

#### Patient questionnaires

The questionnaires (see supplementary data 1) adopted five different types (Information, Category, List, Scale, Open-ended) of questioning styles to elicit specific responses as well as allow the participants the liberty to express themselves as much as possible [10]. The researcher spoke to patients after they have gone through their respective test sampling about sharing their individual experiences as part of the research. Those who agreed and gave written consent were ushered into a staff rest room where questionnaires were filled and handed back to the researcher; the researcher was always on standby for clarification of any item on the questionnaire. Only consecutively consenting OPD patients were recruited for the study. For individuals who were not able to read, the questionnaire items were translated into the native language; the researcher subsequently filled the questionnaires based on participant responses. Although the questionnaire took 5–10 min to complete, there was a low response rate as most of the patients felt they were already tired as a result of perceived stress by the processes in the hospital and were therefore unwilling to participate in the study. More than 90% of individuals who took questionnaires away to return them later never did. Overall, 89 patients (45 from UCC and 44 from Ewim Polyclinic) participated in the study.

Since patients were recruited immediately after they had accessed services at the laboratories, this eliminated the possibility of memory loss with passage of time and strengthened the validity of the findings. The measures employed in the data collection process ensured that the findings closely resembled the everyday patient realities as much as possible. Questionnaire items specifically sought to:

1. find out the pre-sampling information that laboratory scientists (LS) provide to their clients using semi-structured questionnaire.
2. find out the exact information that the LS provide to their clients post-sampling using semi-structured questionnaire.
3. find out from patients how much they know about their respective laboratory tests.
4. explore patients’ perception of who has the responsibility to communicate information about their laboratory testing to them.

#### Laboratory professional questionnaire

The laboratory professional questionnaires (see supplementary data 2) were administered as the last leg of the data collection. Only the laboratory professionals who gave informed consent were recruited for the study.

### Data management

Questionnaire responses were entered into Microsoft Excel 2016. Data was imported into Statistical Package for Social Scientists (SPSS) version 25 (IBM Inco, USA) for analyses.

## Results

### Part 1: observational arm

As the aim of the present study was to assess the laboratory professional-patient interaction as the patient journeyed through the laboratory, only the pre-analytical phase of the laboratory processes that directly involved the patients are described.

### Part 1a: summary of observations of laboratory practitioner-patient interaction at UCC hospital

Generally, patients submit their medical laboratory request form at the documentation section of the laboratory; if the request include stool and/or urine examination, patients are provided with containers to fetch these and return to the documentation section. Once the documentation is done and identification number issued to the requisition form, patients are called into the phlebotomy section over a public address system. The phlebotomy is a small room with two sampling stations manned by two laboratory professionals (LPs). For individuals undergoing tests that require special patient preparation (e.g. semen analyses), these individuals are invited into a dedicated room within the laboratory unit for counselling. Generally, unless a patient is to undergo testing that require fasting status, in which case the LP inquire about dietary status, patients are offered a seat, told to offer the arm/finger for blood sample draw. Patient identification is limited to only name confirmation. Post-sampling care is not standardized and is variable per the LP that attends to a patient. Some LPs ensure that blood clots and apply plaster to the phlebotomy site before the patient leaves the phlebotomy room; other LPs ask patients to leave the phlebotomy unit, with instruction to apply pressure at the site of phlebotomy while sitting in the waiting area.

### Part 1b: summary of observations of laboratory practitioner-patient interaction at Ewim polyclinic

Patients drop their laboratory request form into a box stationed at the front of the laboratory unit; these forms are retrieved by the laboratory unit for documentation. The phlebotomy section is just by the table meant for documentation. Patient go to the phlebotomy unit by responding to their names, which serves as the only means of identification. Pre-sampling information is limited to whether patient has eaten, for samples that require fasting status. Generally, patients are offered a seat, and told to offer the arm for blood sample taking. After sampling, patients are told to sit in the waiting area until when called for their results. In cases where patients are undertaking blood chemistries, they are informed to return next day for results.

### Part 2: patient questionnaire data

The socio-demographic information of patient from the two hospitals are presented in Table 1. Overall, participants from UCC were younger than 49 years vs 15.9% of participants from Ewim polyclinic who were > 49 years. The participants were predominantly females in both facilities (64.4% vs 77.3% respectively from UCC and Ewim polyclinic. Participants with tertiary education constituted a slight majority at UCC hospital compared to majority secondary education attainment at Ewim polyclinic participants.

**Table 1:** Socio-demographic data of participants

Variable	UCC hospital	Ewim Polyclinic
**Age (years)**		
18–20	1 (2.2)	3 (6.8)
21–29	18 (40.0)	11 (25.0)
30–39	17 (37.8)	15 (34.1)
40–49	9 (20.0)	8 (18.2)
50–59	0 (0.0)	4 (9.1)
≥ 60	0 (0.0)	3 (6.8)
**Sex**		
Female	29 (64.4)	34 (77.3)
Male	16 (35.6)	10 (22.7)
**Highest educational attainment**		
No formal education	1 (2.2)	3 (6.8)
Primary	1 (2.2)	4 (9.1)
JHS	6 (13.3)	0 (0.0)
Secondary	10 (22.2)	24 (54.5)
Tertiary	23 (51.1)	9 (20.5)
Vocational	4 (8.9)	4 (9.1)

*JHS* Junior high school

Majority of patients that attended UCC hospital (62.2%) or Ewim polyclinic (81.8%) reported not knowing the specific laboratory testing they were to undergo (Table 2). Also, 48.9% of patients that attended UCC hospital laboratory reported that laboratory staff gave no information prior to sampling compared to 34.1% of patients that attended Ewim polyclinic laboratory. However, 73.3% of patients at UCC hospital laboratory (versus only 25.0% of Ewim Polyclinic patients) reported knowing the turnaround time. Furthermore, whereas a slight majority of patients at the UCC hospital laboratory reported receiving explanation about their laboratory testing from their prescribers, only 6.8% of Ewim Polyclinic patients received such explanation from their prescribers. Moreover, whereas 69.6% (16/23) of patients accessing UCC hospital laboratory stated that their prescribers explained their laboratory testing to them, only 11.1% (1/9) of patients with tertiary education who accessed Ewim Polyclinic laboratory services indicated receiving such explanations from their test requesters. When patients were asked to state the exact laboratory testing requested by their respective practitioner, one wrote: *“I am to have a urine test”* (a 22-year-old female University student at UCC hospital). This client was scheduled for urine routine examination and urine culture and sensitivity testing as stated on her laboratory request form. Also, another patient wrote: *“I am to check my blood level as I don’t feel well”.* (a 40-year-old male client at Ewim Polyclinic). When cross-checked with his laboratory request form, this patient was scheduled for complete blood count and malaria parasite test by blood film.

**Table 2:** Patient knowledge of laboratory testing and turnaround time

Questionnaire item	UCC hospital N (%)	Ewim Polyclinic N (%)	Total N (%)
**Do you know the specific laboratory tests you are to undergo?**			
No	28 (62.2)	36 (81.8)	64 (71.9)
Yes	17 (37.8)	8 (18.2)	25 (28.1)
**Tell us the exact information that the laboratory staff gave you prior to sample taking**			
Requested for permission to take blood and/or to bring urine/stool	23 (51.1)	29 (65.9)	52 (58.4)
No information given	22 (48.9)	15 (34.1)	37 (41.6)
**Do you know how long it is going to take for your results to be ready?**			
No	12 (26.7)	33 (75.0)	45 (50.6)
Yes	33 (73.3)	11 (25.0)	44 (49.5)
**Did your doctor/laboratory test requester explain the laboratory test you are scheduled to undergo to you?**			
No	22 (48.9)	41 (93.2)	63 (70.8)
Yes	23 (51.1)	3 (6.8)	26 (29.2)

When participants were asked about who has responsibility to explain requested laboratory testing, majority (68.9%; 62.2% general OPD and 6.7% ANC cases) of patients that accessed UCC hospital laboratory services stated this was the sole responsibility of the test requester compared to only 31.8% of laboratory attendants at Ewim polyclinic (Table 3). However, 22.2 and 36.2% of participants that accessed laboratory services at UCC and Ewim Polyclinic respectively indicated that explaining laboratory testing was the joint responsibilities of test requester and the laboratory staff. Whereas majority (75.6%) of patients that accessed services at UCC hospital laboratory gave higher than average score on satisfaction scale, almost half (43.2%) of patients that accessed Ewim Polyclinic laboratory gave an average satisfaction score.

**Table 3:** Participants view of responsibility for explanation for laboratory testing

Questionnaire item	UCC hospital; N (%)	Ewim Polyclinic; N (%)
**In your opinion who has responsibility to explain your laboratory testing to you?**		
Doctor	28 (62.2)	7 (15.9)
Doctor and laboratory staff	9 (20.0)	12 (27.3)
Doctor and nurse	0 (0.0)	11 (25.0)
Midwife and laboratory staff	1 (2.2)	4 (9.1)
Midwife	3 (6.7)	7 (15.9)
Laboratory staff	3 (6.7)	3 (6.8)
**On a scale of 1–5, rate your satisfaction with the depth of communication that took place between you and the laboratory staff**		
Excellent (5)	18 (40.0)	9 (20.5)
Very good (4)	16 (35.6)	9 (25.0)
Good (3)	9 (20.0)	19 (43.2)
Satisfactory (2)	1 (2.2)	5 (11.4)
Less than satisfactory (1)	0 (0.0)	2 (4.5)

### Part 3: MLS questionnaire responses

Overwhelming 93.3% of the laboratory professionals indicated the need for the patient to be informed of the laboratory testing turnaround time (Table 4). Whereas 60% of the laboratory professionals intimated that prescribers were solely responsible for informing patient about the specifics of the laboratory test requests, 20% indicated that this should be a shared between prescribers and laboratory professionals. A slight majority of the laboratory professionals reported barriers that hinder effective practitioner-patient communication. One laboratory scientist said: “*the workload is heavy; you feel tired at some point and just want to get to next patient”.* (Male laboratory professional with 6 years’ work experience, at UCC hospital). Another laboratory scientist also wrote that: “*it always stresses me to see many patients in the waiting area and the complains they make; I try to spend as little time on each patient as possible so we could clear them. Even if you decide to have lengthy discussion with the patient, it is in the hearing of all, so what is the point”.* (Female laboratory professional with 4 years laboratory experience at Ewim Polyclinic).

**Table 4:** Laboratory professional responses to questionnaire items

Questionnaire item	Facility		Total
Facility	Ewim	UCC	
**Sex**			
Female	2	3	5 (33.3)
Male	3	7	10 (66.6)
**Grade/Cadre**			
Medical Lab. Scientist	2	6	8 (53.3)
Senior Lab. Technologist	0	3	3 (20.0)
Lab Assistant	3	1	4 (26.7)
**Who has responsibility to inform patients about laboratory tests**			
Prescriber only	4	5	9 (60.0)
Prescriber and laboratory personnel	1	2	3 (20.0)
Laboratory personnel only	0	3	3 (20.0)
**Does the patient need to know the laboratory testing turnaround time?**			
No	0	1	1 (6.7)
Yes	5	9	14 (93.3)
**Are there barriers to effective communication in your facility?**			
No	2	5	7 (46.7)
Yes	3	5	8 (53.3)

## Discussion

There have been extensive studies on patient turnaround times at various units in the hospital settings. However, there is scarcity of studies that actually explored real-time patient experiences at the laboratory section/department of hospitals in sub-Saharan Africa. This study thus sought to use real time observations of patient-laboratory practitioner interactions to provide empirical data regarding actual lived patient experiences at the laboratory units of two hospitals and explored the depth of pre- and post-sampling information provided to patients by laboratory professionals. This study found a discrepancy between the actual experiences of patients (as observed by the researcher as well as stated from the patient perspective) and information given by the laboratory professionals concerning the communication of the turnaround times to patients. This study also found a general lack of standard operating procedures to standardize LP-patient interaction resulting in variability in communication between LP and patients. Moreover, patients undertaking blood chemistries are routinely asked to return the following day for laboratory results which has the potential to impede prompt medical decision making.

Although, an overwhelming 93.3% of laboratory professionals intimated the importance of communicating the testing turnaround time to the patients, only half of patients (50.6%; 45/89 patients) indicated knowing when their respective test results were going to be ready. Thus, although the laboratory professionals acknowledged the need to communicate testing turnaround time to patients, these are not routinely communicated to patients in the course of work. This was duly acknowledged by some laboratory professionals who variously referred to the pressure from high workload as being a major barrier to effective patient communication. Indeed, what was stated by the patients more reflect the realities of the patient-practitioner communication as it conforms to the researcher’s observational data. The 50.6% of patients who were informed about the testing turnaround time as found by this study is however lower than the 88% reported in a national survey in Ethiopia [11]. Incidentally, majority of the individuals who reported knowing their respective testing turnaround times were those undertaking blood chemistries. It is important to note that irrespective of the time in the day in which the patient accessed laboratory services, with the exception of fasting blood glucose which was undertaken using rapid diagnostic test kit, all those undertaking blood chemistries were always informed to return the next day for results. Even though the LPs indicated this to be the status quo owing to the relatively longer time it took to complete one cycle of testing on the blood chemistry analyser, this researcher is of the view that this practice needs re-consideration as it does not auger well for prompt medical decision making. Taken together with the supposition that about 70% of clinical decisions are estimated to be based on laboratory results [9], quality of laboratory services at the two hospitals is questionable as the timeliness aspect of quality is not routinely addressed.

Interestingly, although 60% of the LPs stated that informing patients about the specifics of their laboratory testing was the sole prerogative of the respective test requester, only 29.2% (26/89) of patients indicated such explanations were given by their test requesters. It is important to note that a greater proportion of the patients that were informed by their test requester about the laboratory testing specifics were from the UCC hospital which serves mainly tertiary students. Although this study neither collected data from the perspective of the requesters of laboratory tests, nor undertake observations of test requester-patient interactions, future studies that address this gap in the present study will provide a comprehensive understanding of the patient-practitioner interaction during hospital visit. This is important considering that if both test requesters and laboratory professionals each make the assumption that the other professional was responsible for explaining laboratory testing, the patient stands to lose from this vital aspect of clinical service. In the absence of such data, the findings reported herein strongly argues for the need for engagement of all practitioners directly involved in either requesting or undertaking laboratory assays for patients to clarify responsibilities. This is important considering that a previous study in Tanzania found associations between inadequate explanations regarding laboratory procedures and anxiety in patients [12].

Interestingly, LPs’ and patient engagement during sample taking at the study sites is reduced to the barest minimum. For example, despite recommendations that patient identification should be established using a minimum of three identifiers [13], patients were identified at both laboratories only by their respective names; no recourse to other means of establishing patient identity are explored at the two laboratory units. The existing work paradigm is therefore prone to patient misidentification. Besides, none of the laboratories has written standard operating procedures (SOPs) regarding LP-patient interaction. Clinical laboratory practice is globally regulated by the use of SOPs that ensures standardization of practice [14]. In the absence of such SOPs, whatever constitutes an appropriate level of care is left to the discretion of the professionals with its attendant practical implications. Thus, there are wide variations in the services offered to patients even within the same facility. For example, whereas some LPs ask patients to leave the phlebotomy unit with instruction to apply pressure on a piece of cotton applied to the phlebotomy site, other LPs ensured that blood flow has ceased at the phlebotomy site, apply adhesive to cover the open wound before asking the patient to leave. As a consequence of this, it is not uncommon to find blood-stained pieces of cotton littered at the patient waiting area with its attending biohazard risks. These variations in practice are so widespread that even protocols for managing blood spills are variable; for example, whereas other professionals use alcohol to clean blood spills, other professionals rather use bleach to do same. Even though this study was limited to the two hospitals’ laboratories and may therefore be premature to generalize the findings to other facilities, it is plausible to suppose that this might be happening in other laboratories and therefore needs holistic approach to ensure standardization of patient care. Although it can be argued that the existence of SOPs will not automatically lead to elimination of all forms of practice variations, this researcher is of the view that the implementation of SOPs will dramatically reduce these variations in care and assure consistency in patient care delivery in the laboratory units. A critical avenue that enables health professional to update their practices is through contextually relevant continuous professional development (CPD) programmes [15, 16]. Thus, the national association of the laboratory professionals should consider engaging relevant stakeholders to facilitate such CPD seminars to begin the process of standardization of patient-laboratory professional interactions.

Furthermore, this study found that patients generally have poor knowledge about their scheduled laboratory testing. Overall, only 28.1% of patients stated knowing the specifics of their respective laboratory testing. When patients were asked to respectively give the names of the exact laboratory testing, less than 15% could correctly identify their respective testing. For example, some patients indicated that they were to undertake urine test; when these were compared to their respective requisition sheets, some were to undergo both urine routine examination and urine culture. Thus, these patients knew only of the laboratory test in general terms. Even though a higher proportion of patients accessing laboratory services at UCC hospital had tertiary education, only about a third of these patients stated knowing their respective laboratory testing (compared to 18.2% Ewim Polyclinic laboratory patients). Such poor understanding on the part of the patients may therefore not be necessarily a function of educational attainment of patients. This is an area that must be addressed by a concerted effort between healthcare professionals to ensure better patient understanding of laboratory testing and respective procedures. Future studies should consider patients accessing care at other departments of these hospitals for a comprehensive public health engagement initiative through systems thinking approaches.

It is important to point out that certain organisational factors and constraints at the two study sites prevented adequate patient-practitioner interactions. For example, a conducive environment that assures privacy and confidentiality is required for effective LP-patient interaction [17, 18]. However, it is near impossible to attain either confidentiality or privacy at the two study sites. At Ewim Polyclinic, the documentation and phlebotomy sections of the laboratory unit are one and the same room; the waiting area is eavesdropping away. Consequently, whatever is discussed between the LP and patient is in the hearing of other patients in the waiting area. Although the waiting area at UCC laboratory was separate from the phlebotomy unit, the two phlebotomy stations are in the same small room. Thus, two patients being attended to gets to hear whatever the other patient is being offered. As space is a major constraint in most laboratories in sub-Saharan Africa [19], architectural designs of future hospitals should give adequate priority to the set-up of laboratories to ensure adequacy of laboratory practitioner-patient interactions.

There was a general disconnect between what laboratory staff stated in questionnaire responses as their standard patient care, and what was observed first-hand by the researcher. In the patients’ questionnaire responses, 41.6% of the patients stated that no information was given by the laboratory personnel prior to sampling. This agrees with the researcher’s observational data, but was contrary to responses provided by the laboratory professionals. One of the key strengths of the present study is collection of observational data which provided a priceless perspective in contextualizing the findings of this study and offered insight into the apparent disparity between the laboratory professionals and patient responses. The questionnaire responses by the laboratory professionals are suggestive that the laboratory professionals have the theoretical knowledge of what constitutes adequate practitioner-patient interactions. However, why they fail to actualize this knowledge when attending to patients require further investigation. Among the reasons offered by the laboratory professionals include the issue of heavy workload that makes it more demanding to effectively communicate with clients. With UCC and Ewim polyclinic laboratories averagely respectively attending to 94 and 62 patients/day, this heavy workload may be an important consideration in any attempt made to address adequacy of patient-practitioner interactions.

The major limitations of this study include the small sample size and the fact that the study was limited to the laboratory units of two hospitals. Even though the study had originally intended a larger sample size and additional hospitals, the COVID-19 pandemic and the subsequent global shut down restricted the sampling period. Notwithstanding these limitations, the methodological triangulation employed for data collection has increased the scientific rigour of the data reported herein and could thus be used to inform future studies as Ghana gradually returns to normalcy. As stated by other researchers [10, 11], one of the key limitations of data gathering through questionnaires is social desirability bias where participants may give responses based on theoretical understanding of concepts. It is therefore not uncommon for questionnaire-based studies exploring patient hospital experiences to produce results that may be far removed from realities of actual patient experiences. In spite of this obvious limitation of the use of questionnaire, the use of observational data collection enabled the researcher to reconcile any apparent discrepancy between data collected from patient and laboratory professional perspectives. This researcher however acknowledges that the shear presence of the researcher at the sampling unit has the potential to influence participant behaviour as such realization by the LP and/or patient may subconsciously bias their respective behaviour. However, the observation was necessary as it provided crucial data that could not have been obtained otherwise.

## Conclusion

The two laboratories should employ SOPs to improve patient education and standardize practitioner-patient interactions.

## Supplementary Information

**Additional file 1: Supplementary data 1.** Patient questinnaire. **Supplementary data 2.** Questionnaire for laboratory staff only.

## Data Availability

The dataset used and/ or analysed during the current study is available from the corresponding author upon reasonable request.

## Abbreviations

SOPs: Standard operating procedures
LP: Laboratory professionals
CPD: Continuous professional development
