# Supplementary material for: A cross-case analyses of laboratory professionals-patients interaction for patients accessing laboratory services at University of Cape Coast hospital and Ewim Polyclinic in the Cape Coast Metropolis, Ghana
Source: BMC Health Serv Res. 2021 May 28;21:520. doi: 10.1186/s12913-021-06560-8 (PMC8160390; doi:10.1186/s12913-021-06560-8)
Supplement: Supplementary file 1 — Additional file 1: Supplementary data 1. Patient questinnaire. Supplementary data 2. Questionnaire for laboratory staff only. [file 12913_2021_6560_MOESM1_ESM.docx]

Dear Sir/Madam

We are interested in learning about your opinion regarding the communication about your laboratory testing anytime you visit the hospital. The researcher (Patrick Adu) is a staff of University of Cape Coast, but is undertaking this research as part of postgraduate study at the Lancaster University, UK. The findings of the study shall be useful in the identification of any potential gap in communication between healthcare practitioners and patients. Therefore, the findings may be published in international journal to increase access to the findings.

As the study is voluntary, your participation very much appreciated. Your responses shall be kept anonymous and confidential. You are being approached because you just accessed service at the laboratory unit of the hospital, or a laboratory staff of the hospital. The questionnaire will take between 5 – 10 minutes of your time. You may reach the lead researcher on +233 50 460 7803 or via email at Patrick.adu@ucc.edu.gh.

Thank you for participating in the study.

**SUPPLEMENTARY DATA 1: PATIENT QUESTINNAIRE**

Path #:

**PART A: GENERAL INFORMATION**

1. Please select which of the following best describes your age in years

[ ] 18 – 20 [ ] 21 – 29 [ ] 30 – 39 [ ] 40 – 49 [ ] 50 – 59 [ ] 60+

1. Sex [ ] male [ ] female
2. Patient type

[ ] OPD [ ] In-patient

1. Please select which of the following best describes your highest educational attainment?

[ ] No formal education [ ] Primary [ ] Secondary [ ] Vocational [ ] Tertiary

**PART B: INFORMATION ABOUT YOUR LABORATORY TESTING**

1. Please indicate what **specimen/sample** you were required to give (tick as many as applicable)

[ ] Stool [ ] Urine [ ] sputum [ ] blood [ ] others, please specify________________

1. Do you know what specific test(s) was requested in your case?

[ ] Yes [ ] No

If **yes**, please specify ………………………………………………………….

1. Tell us what information the laboratory staff gave you **before** drawing your sample ……………………………………………………………………………………………………………………………………………………………………………………………………………………………………………………………………………………………..
2. Tell us what exact information the laboratory staff gave you **after** drawing you sample ………………………………………………………………………………………………………………………………………………………………………………………………………………………………………………………………………………………………………………………………………………………………………………………………
3. Do you have an idea concerning **how long** it is going to take before your laboratory results will be ready?

[ ] Yes [ ] No

If **yes**, please specify……………………………

1. Did the doctor explain the test(s) you were to undertake?

[ ] Yes [ ] No

1. In your opinion, who has the responsibility to explain the test you are scheduled to undertake? (tick as many as applicable)

[ ] Doctor [ ] nurse [ ] laboratory staff [ ] others, please specify …………………………

1. On a scale of 1 – 5, how would you rate your experience of the standard of communication regarding your laboratory testing in connection with the following staff

| Staff | 5 (excellent) | 4 (very good) | 3 (good) | 2 (satisfactory) | 1 (less than satisfactory) |
| --- | --- | --- | --- | --- | --- |
| Doctor |  |  |  |  |  |
| Nurse |  |  |  |  |  |
| Laboratory |  |  |  |  |  |

**SUPPLEMENTARY DATA 2: QUESTIONNAIRE FOR LABORATORY STAFF ONLY**

**PART 1: GENERAL INFORMATION**

Path #:

1. Age

[ ] 18 – 20 [ ] 21 – 29 [ ] 30 39 [ ] 40 – 49 [ ] 50 - 59

1. Sex

[ ] male [ ] female

1. Grade/cadre

___________________________________

1. Please tell us how long you have been in practice

_____________________________________________

**PART 2: INFORMATION ABOUT YOUR INTERACTION WITH THE PATIENT**

1. What standard information do you give to your clients before sampling?

____________________________________________________________________________________________________________________________________________________________________________________________________________________________________________________________________________________

1. What standard information do you give to your clients after sampling?

____________________________________________________________________________________________________________________________________________________________________________________________________________________________________________________________________________________

1. In your opinion, who has the responsibility to explain what laboratory tests a patient is requested to undertake?

__________________________________________________________________________________________________________________________________________

1. Does the patient need to know the time lag on the specific test(s) that has been requested?

[ ] yes [ ] no

1. Please explain your choice in 8 above

_______________________________________________________________________________________________________________________________________________________________________________________________________________

1. Are there barriers to effective practitioner-patient communication in this facility?

[ ] Yes [ ] No

1. If yes, tell us more about them

____________________________________________________________________________________________________________________________________________________________________________________________________________________________________________________________________________________
